# Supplementary material for: The Synergistic Impact of Crystal Seed and Fluoride Ion in the Synthesis of Silicalite-1 Zeolite in Low-Template Systems
Source: Materials (Basel). 2024 Jan 4;17(1):266. doi: 10.3390/ma17010266 (PMC10779753; doi:10.3390/ma17010266)
Supplement: Supplementary file 1 [file materials-17-00266-s001.zip › materials-2768914-supplementary.pdf]

Supplementary Information for

The synergistic impact of crystal seed and fluoride ion in the synthesis of Silicalite-1 zeolite in low-template system

Xiaojing Meng [1,*], Yanjie Qin [1], Yang Zhang [1], Min Li [1], Huibang Huang [1], Jiaqin Peng [1], Liangxu Zhou [1], Jian Feng [1]

(1. College of Chemistry and Chemical Engineering, Chongqing University of Science and Technology, Chongqing 401331, China.* Corresponding author)

E-mail address: mengxiaojing88@126.com

Figure Captions

Figure S1. XRD patterns of Silicalite-1 zeolites with different F/Si ratios: (a) 0, (b) 0.06, (c) 0.1, (d) 0.15, (e) 0.2.

Figure S2. SEM images of Silicalite-1 zeolites with different F/Si ratios: (a) 0, (b) 0.06, (c) 0.1, (d) 0.15, (e) 0.2, (f) 0.2 (High magnification).

Figure S3. XRD patterns of Silicalite-1 zeolites with different S-1 seeds contents: (a) 1 wt%, (b) 5 wt%, (c) 10 wt%, (d) 20 wt%, with a F/Si = 0.1.

Figure S4. High-resolution SEM images of Silicalite-1 zeolites with a F/Si of 0.1 under different S-1 seeds contents: (a) 1 wt%, (b) 5 wt%, (c) 10 wt%, and (d) 20 wt%.

Figure S5. XRD patterns of Silicalite-1 zeolites (S-1 seeds = 10 wt%) with different F/Si ratios: (a) 0.03, (b) 0.05, (c) 0.1, (d) 0.3, (e) 0.4.

Figure S6. SEM images of Silicalite-1 zeolites with different F/Si ratios: (a) 0.03, (b) 0.05, (c) 0.3, and (d) 0.4, with S-1 seeds = 10 wt%.

Figure S7. SEM images of Silicalite-1 zeolites with different crystallization time in the system containing NH_4F alone: (a) 2 h, (b) 4 h, (c) 6 h.

Figure S8. SEM images of Silicalite-1 zeolites with different crystallization time in the system containing S-1 seeds alone: (a) 2 h, (b) 4 h, (c) 6 h.

Figure S9. XRD patterns of Silicalite-1 zeolites with different crystallization time in the system containing NH_4F and S-1 seeds: (a) 12 h, (b) 24 h, (c) 48 h.

Figure S1.

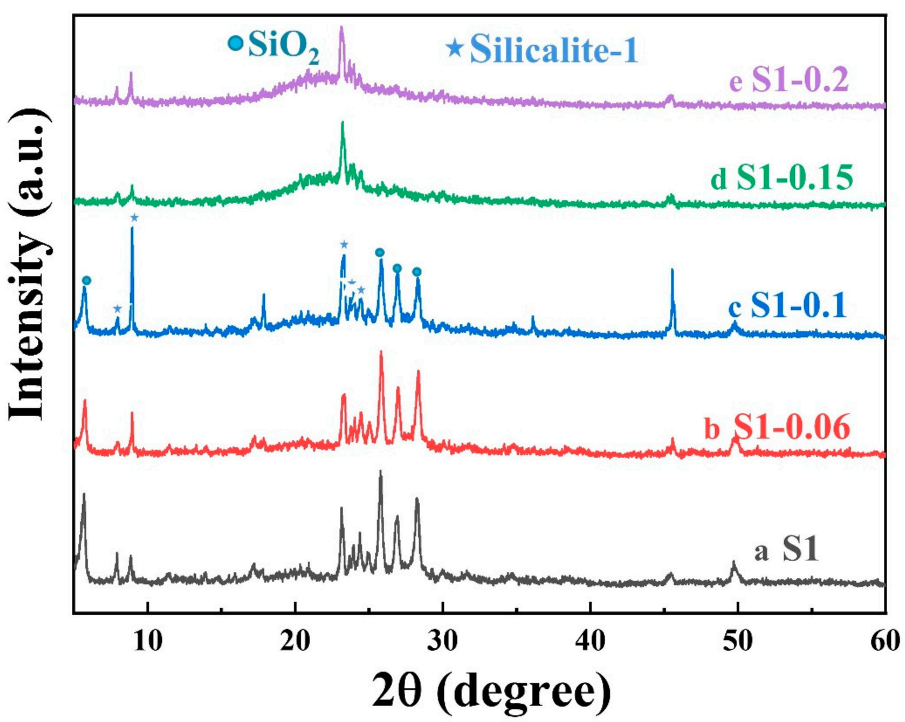

SiO2
Silicalite-1
e S1-0.2
d S1-0.15
c S1-0.1
b S1-0.06
a S1
Intensity (a.u.)
10
20
30
40
50
60
2θ (degree)

Figure S2.

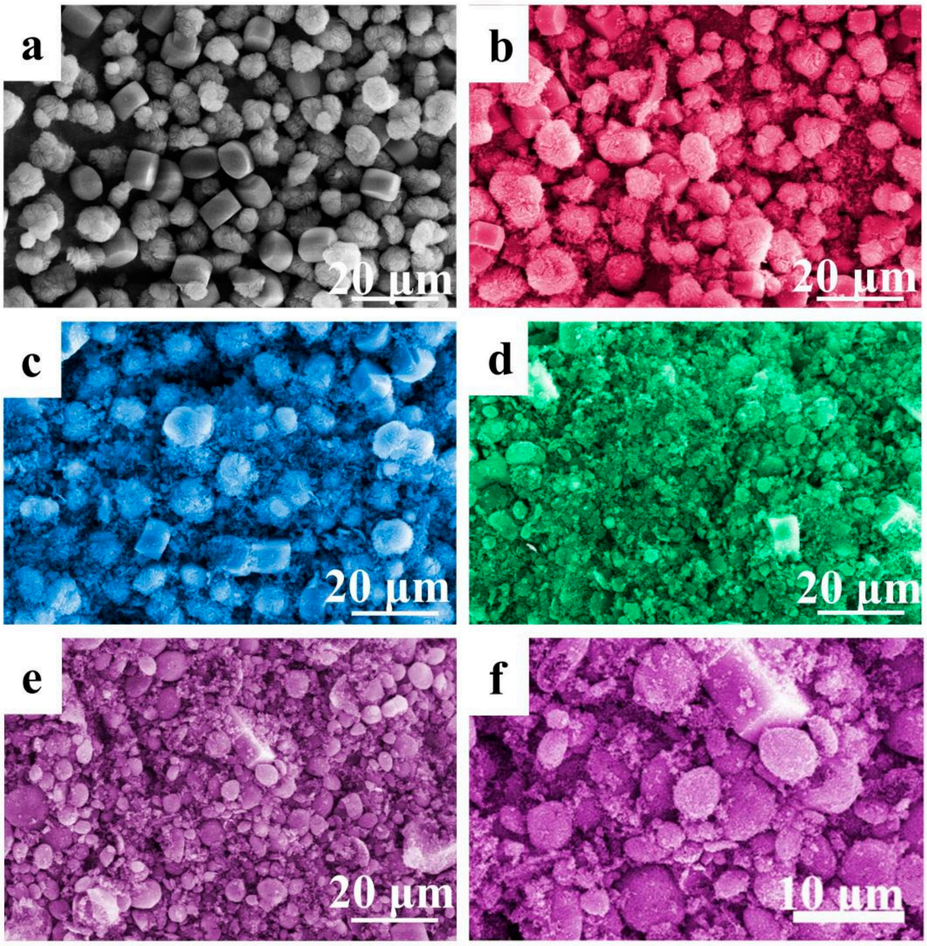

Figure S3.

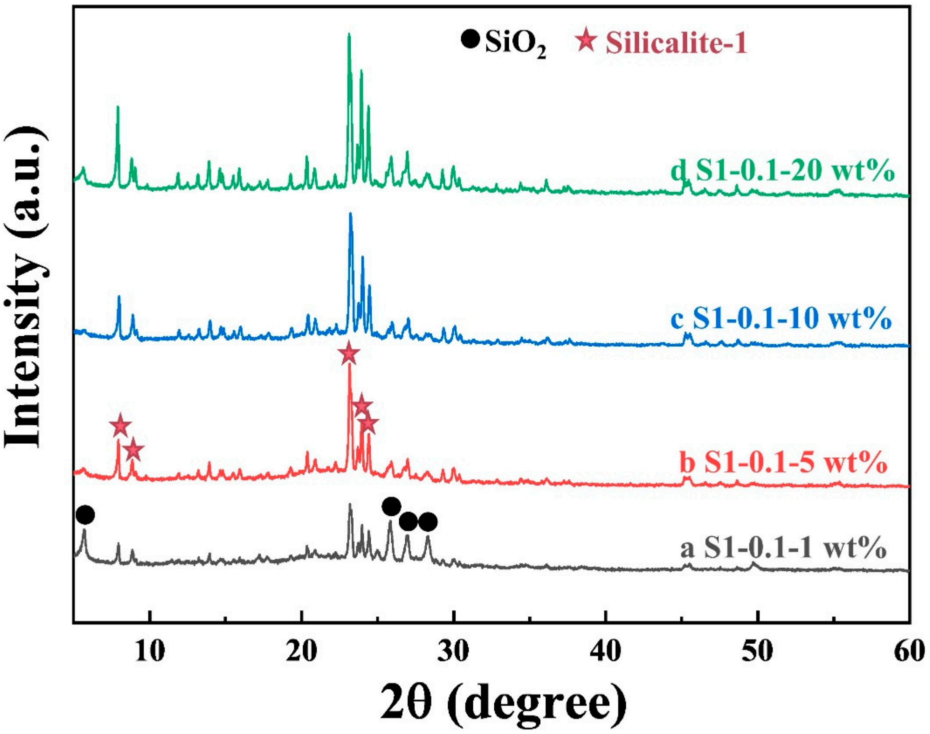

SiO2
Silicalite-1
Intensity (a.u.)
d S1-0.1-20 wt%
c S1-0.1-10 wt%
b S1-0.1-5 wt%
a S1-0.1-1 wt%
10
20
30
40
50
60
2θ (degree)

Figure S4.

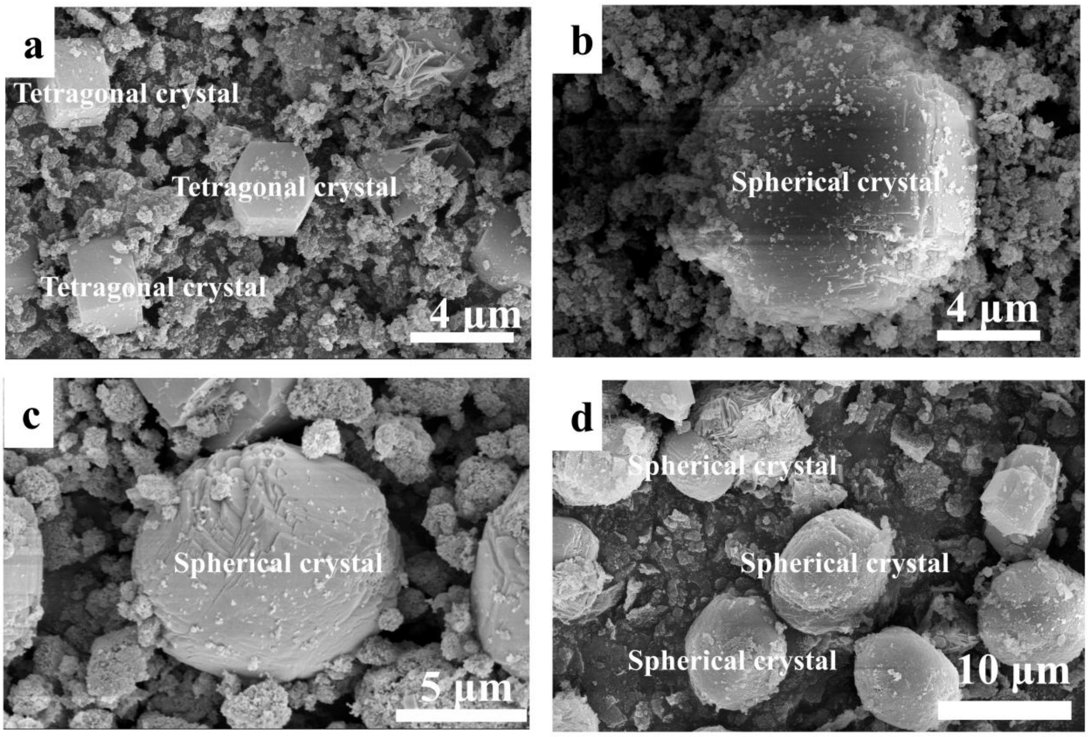

a
Tetragonal crystal
Tetragonal crystal
Tetragonal crystal
4 µm
b
Spherical crystal
4 µm
c
Spherical crystal
5 µm
d
Spherical crystal
Spherical crystal
Spherical crystal
10 µm

Figure S5.

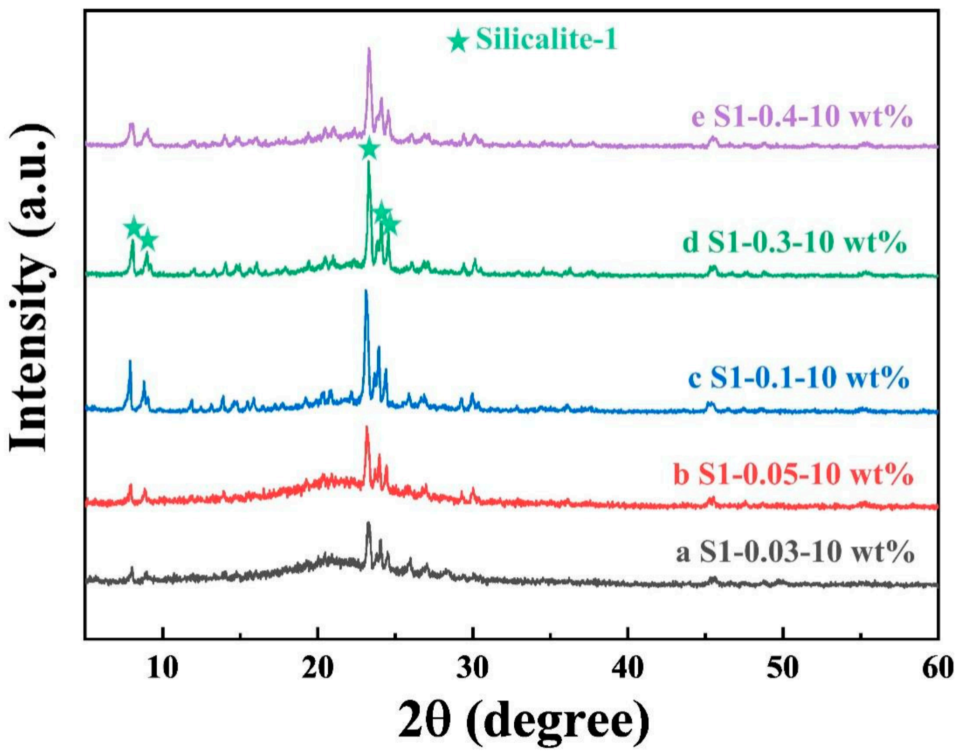
★ Silicalite-1
e S1-0.4-10 wt%
d S1-0.3-10 wt%
c S1-0.1-10 wt%
b S1-0.05-10 wt%
a S1-0.03-10 wt%
Intensity (a.u.)
10
20
30
40
50
60
2θ (degree)

Figure S6.

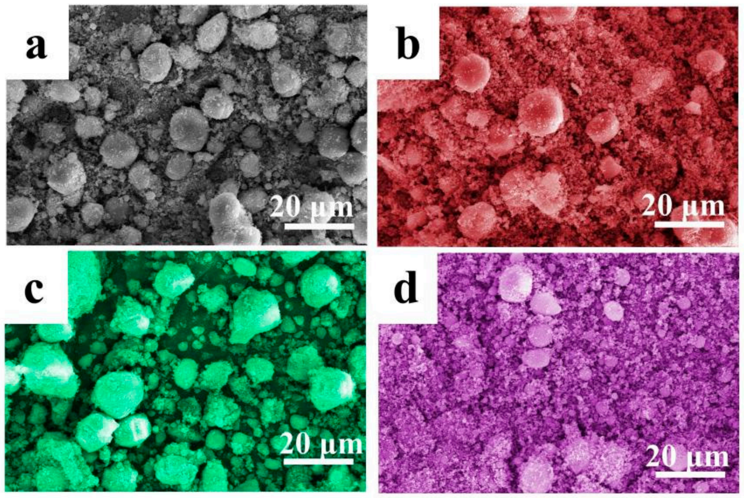

Figure S7.

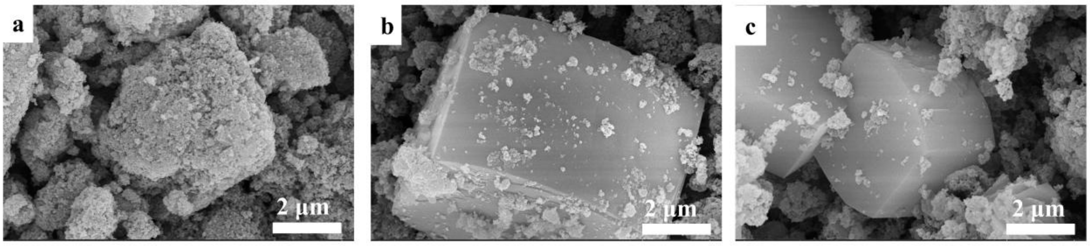

Figure S8.

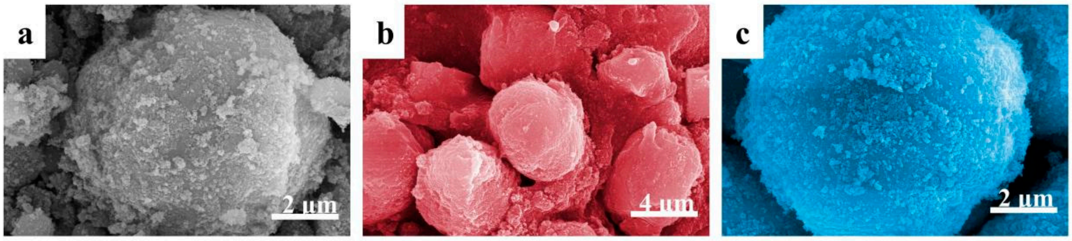

Figure S9.

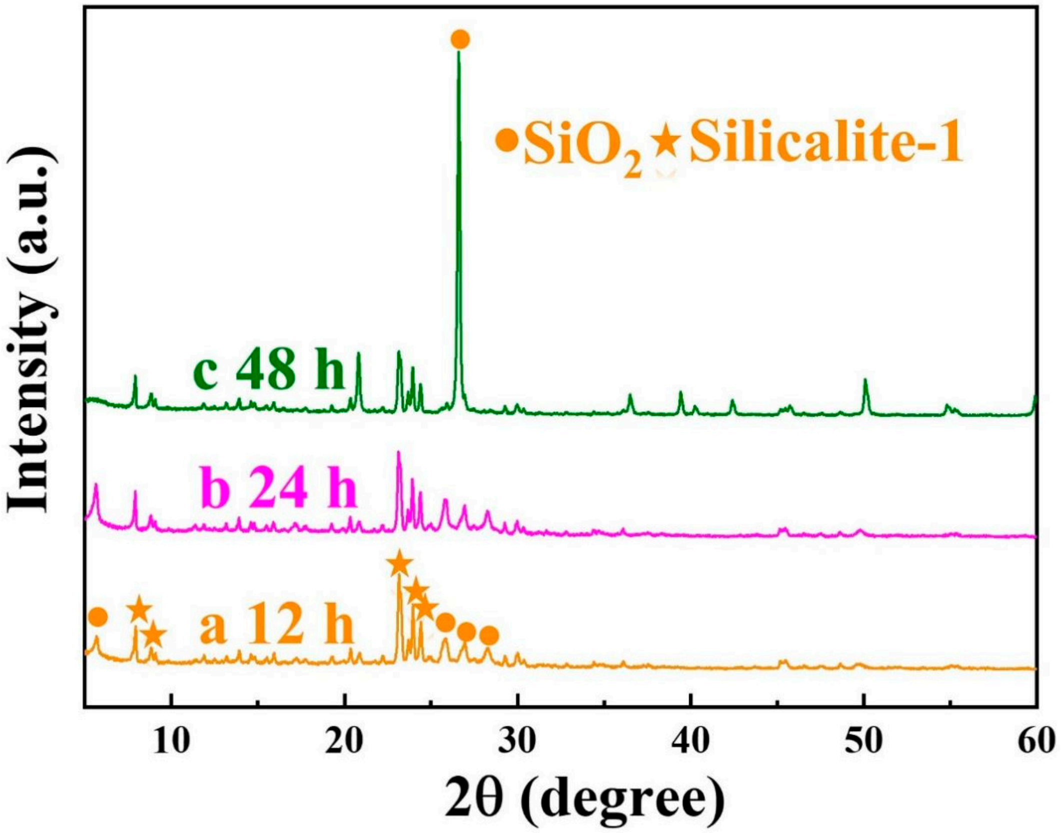
●SiO$_2$ ★Silicalite-1
c 48 h
b 24 h
a 12 h
Intensity (a.u.)
10
20
30
40
50
60
2θ (degree)
